# Illusions of causality: how they bias our everyday thinking and how they could be reduced

**DOI:** 10.3389/fpsyg.2015.00888

**Published:** 2015-07-02

**Authors:** Helena Matute, Fernando Blanco, Ion Yarritu, Marcos Díaz-Lago, Miguel A. Vadillo, Itxaso Barberia

**Affiliations:** ^1^Departamento de Fundamentos y Métodos de la Psicología, Universidad de Deusto, Bilbao, Spain; ^2^Primary Care and Public Health Sciences, King’s College London, London, UK; ^3^Departamento de Psicología Básica, Universitat de Barcelona, Barcelona, Spain; ^4^EventLab, Departamento de Personalidad, Evaluación y Tratamiento Psicológico, Universitat de Barcelona, Barcelona, Spain

**Keywords:** causal learning, cognitive biases, contingency judgment, illusion of causality, illusion of control, science teaching, scientific methods, scientific thinking

## Abstract

Illusions of causality occur when people develop the belief that there is a causal connection between two events that are actually unrelated. Such illusions have been proposed to underlie pseudoscience and superstitious thinking, sometimes leading to disastrous consequences in relation to critical life areas, such as health, finances, and wellbeing. Like optical illusions, they can occur for anyone under well-known conditions. Scientific thinking is the best possible safeguard against them, but it does not come intuitively and needs to be taught. Teaching how to think scientifically should benefit from better understanding of the illusion of causality. In this article, we review experiments that our group has conducted on the illusion of causality during the last 20 years. We discuss how research on the illusion of causality can contribute to the teaching of scientific thinking and how scientific thinking can reduce illusion.

## Introduction

In today’s world, there is a growing tendency to trust personal beliefs, superstitions, and pseudoscience more than scientific evidence (Lewandowsky et al., 2012; Schmaltz and Lilienfeld, 2014; Achenbach, 2015; Carroll, 2015; Haberman, 2015). Superstitious, magical, and pseudoscientific thinking refer to ungrounded beliefs that are not supported by current evidence (Lindeman and Svedholm, 2012). Many of them involve causal illusions, which are the perception of a causal relationship between events that are actually unrelated. Examples of causal illusions can easily be found in many important areas of everyday life, including economics, education, politics, and health. Indeed, causal illusions and related cognitive biases such as overconfidence, the illusion of control, and illusory correlations have been suggested as the basis of financial bubbles (e.g., Malmendier and Tate, 2005), social stereotypes (Hamilton and Gifford, 1976; Crocker, 1981; Murphy et al., 2011), hostile driving behavior (Stephens and Ohtsuka, 2014), social intolerance and war (Johnson, 2004; Lilienfeld et al., 2009), and public health problems such as the increased popularity of alternative and complementary medicine (Matute et al., 2011; Blanco et al., 2014).

For example, many reports have shown that homeopathy has no causal effect on patient health other than a placebo effect (Shang et al., 2005; Singh and Ernst, 2008; Ernst, 2015; National Health and Medical Research Council, 2015). Even so, 34% of Europeans believe that homeopathy is effective (European Commission, 2005). The illusion of causality in this case arises from very simple intuitions based on coincidences: “I take the pill. I happen to feel better. Therefore, it works.” Many people in today’s world have come to believe that alternative medicine is effective and that many practices not supported by evidence are reliable simply because “they work for them,” as they put it. That is, they *feel* as if recovery was caused by the treatment (Lilienfeld et al., 2014). Some people go even further and prefer alternative medicine over scientific medicine. This attitude is causing serious problems for many people, sometimes even death (Freckelton, 2012).

Despite the effort of governments and skeptical organizations to promote a knowledge-based society, the effectiveness of such campaigns has been limited at best (Schwarz et al., 2007; Nyhan and Reifler, 2015). Two in five Europeans are superstitious (European Commission, 2010), and more than 35% of Americans believe in haunted houses or extrasensory perception (Moore, 2005). Superstitious and pseudoscientific thinking appear to be increasing in several European countries (while not changing in others; see European Commission, 2010). This type of thinking often guides health, financial, and family decisions that should be guided by contrasted knowledge and empirical evidence. In the UK, the House of Commons Science and Technology Committee (2010) complained in a recent report that even though the UK Government acknowledges that “there is no credible evidence of efficacy for homeopathy” (p. 42), the UK Government is still funding homeopathy and providing licenses that allow for retail in pharmacies, so that “the Government runs the risk of endorsing homeopathy as an efficacious system of medicine” (p. 42). A similar situation can be found in most countries. In Australia, the National Health and Medical Research Council (2015) recently published a comprehensive report that warns people that “there are no health conditions for which there is reliable evidence that homeopathy is effective” (p. 6). This report goes one step further and attempts not only to provide evidence, but importantly, to educate people on why personal beliefs and experiences are not a reliable source of knowledge and why scientific methods should always be used when assessing causality.

In the age of the Internet, when both science and pseudoscience are at a click’s distance, many people do not know what to believe anymore. Rejection of science seems to be growing worldwide (Achenbach, 2015), and it is becoming increasingly difficult to eradicate myths once they start to spread (Lewandowsky et al., 2012; Schmaltz and Lilienfeld, 2014). There are many possible reasons why people are rejecting science. Finding evidence-based strategies to counteract them should become a priority if we aim to create efficient public campaigns and policies that may change scientific education (Schwarz et al., 2007; Gough et al., 2011). As noted by Bloom and Weisberg (2007), resistance to science is becoming particularly intense in societies where pseudoscientific views are transmitted by trustworthy people. Unfortunately, scientists are not always regarded as the most trustworthy source in society because people often trust friends and family more (Eiser et al., 2009). An excellent example is the anti-vaccine crisis in 2015 and the fact that mainly rich and well-educated parents are deciding not to vaccinate their children despite scientific and governmental alarms (Carroll, 2015).

One of the main difficulties is that showing people the facts is not enough to eradicate false beliefs (e.g., Yarritu and Matute, 2015). This can even sometimes have the opposite effect of strengthening the myth (Lewandowsky et al., 2012; Nyhan and Reifler, 2015). Moreover, teaching scientific methods does not seem to be enough either (Willingham, 2007; Schmaltz and Lilienfeld, 2014), as many naïve preconceptions may survive even after extended scientific training (Shtulman and Valcarcel, 2012). However, learning how to think scientifically and how to use scientific methods should at least provide an important safeguard against cognitive biases (Lilienfeld et al., 2012; Barberia et al., 2013). Indeed, we suggest that scientific methods constitute the best possible tool, if not the only one, that has been developed to counteract the illusion of causality that can be found at the heart of many of those problems. But scientific thinking and scientific methods are not intuitive and need to be taught and practiced. A better understanding of how the illusion of causality works should cast light on how to improve the teaching of scientific thinking and make it more efficient at reducing the illusion. In this article, we review the experiments that our group has conducted on the illusion of causality and discuss how this research can inform the teaching of scientific thinking.

## Illusions of Causality, Contingency, and Scientific Methods

Just as there are optical illusions that make people see things larger than they are or with different shapes or colors, there are illusions of causality that make people perceive that one event causes another when there is just a coincidence between them. Indeed, just as people have no way of assessing size or weight accurately without using specially designed tools (such as measuring tapes or balances), they are likewise not prepared to assess causality without tools. Scientific methods, particularly the experimental approach, are the external tools that society has developed to assess causality.

It could be argued that people often infer size, color, or weight intuitively, and it is true that they can often do so relatively well even in the absence of any external help. However, we all know that people make errors. When facing an important or critical situation in life, such as buying a house or deciding where to land an aircraft, most people will not trust their intuitions but will rely on external aids such as measuring tapes or Global Position System (GPS). By the same reasoning, when trying to assess causality intuitively, people can often be relatively accurate, as demonstrated in many experiments (e.g., Ward and Jenkins, 1965; Peterson, 1980; Shanks and Dickinson, 1987; Allan, 1993; Wasserman et al., 1993). At the same time, however, it has also been shown in countless experiments that under certain well-known conditions, people can make blatant errors when judging causal relations with the naked eye (Alloy and Abramson, 1979; Allan and Jenkins, 1983; Lagnado and Sloman, 2006; Msetfi et al., 2007; Hannah and Beneteau, 2009; Blanco et al., 2014). Even scientists, who are used to thinking critically and applying rigorous methodologies in their work, have sometimes been found to develop superstitions in their daily life (Wiseman and Watt, 2006; Hutson, 2015) and to forget that causality cannot be accurately assessed based on quick intuitions (Kelemen et al., 2013; Phua and Tan, 2013).

Examples of intuitive assessment of causality (sometimes correct, sometimes biased) can be found easily in everyday life. A company could initiate a new training program, attract more clients, and assume that the new program was effective. Upon carrying a good-luck charm and playing a fantastic game, one cannot avoid feeling that the charm had a critical role in victory. This tendency to detect causal relationships is so strong that people infer them even when they are rationally convinced that the causal mechanism that would make the relationship plausible does not exist. As another example, your favorite sports team might lose the game just as you go to the kitchen for a moment. Despite knowing that a causal relation does not exist between your behavior and the outcome of the game, feeling as if you were somewhat responsible for that failure can be hard to avoid (Pronin et al., 2006).

To counteract the illusion of causality, it is essential to understand that the illusion is not a matter of intelligence or personality (Wiseman and Watt, 2006). Illusions of causality can occur for anyone, just like visual illusions. They occur because of the way the human mind has evolved: It extracts causality from coincidences. Thus, counteracting the illusion is a matter of being able to use the right tools and knowing when and how to use them. We cannot think of a better safeguard against the illusions of causality than scientific thinking, which involves skepticism, doubt, and rigorously applying scientific methods, particularly the experimental approach.

The basic idea in causal judgment research is that people often need to infer whether a relationship is causal through observing ambiguous cases and incomplete evidence. In the simplest causal learning situations, there are two events—a potential cause and an outcome—that can be repeatedly paired over a series of trials. Four different types of trials can result from the possible combinations of the presence or absence of these two binary events. Both the potential cause and the outcome can occur (type *a* trials), the cause may occur while the outcome does not (type *b* trials), the cause may not occur and the outcome still occurs (type *c* trials), and finally, neither the cause nor the outcome may occur (type *d* trials). These four trial types are shown in the contingency matrix in Table 1.

**TABLE 1:** **Contingency matrix showing the four different types of trials as a function of whether or not the cause and the outcome are present**.

****	**Outcome present**	**Outcome absent**
Cause present	a	b
Cause absent	c	d

The Δ*p* index (Allan, 1980) has been broadly accepted as a normative measure of contingency on which participants’ subjective estimations of causality should be based (e.g., Jenkins and Ward, 1965; Shaklee and Mims, 1981; Allan and Jenkins, 1983; Shanks and Dickinson, 1987; Cheng and Novick, 1992). This index is computed as the probability that the outcome occurs in the presence of the potential cause, P(O|C), minus the probability that it occurs in its absence, P(O|¬C). These probabilities can easily be computed from the number of trials of each type (*a, b, c, d*) in Table 1:

Δp=P(O|C)−P(O|¬C)=[a/(a+b)]−[c/(c+d)].

When an outcome’s probability of occurrence in the presence of a cause is larger than that without the cause, Δ*p* is positive. This means that the potential cause contributes to producing the outcome. For instance, if the probability of recovery from a given disease is larger when people take a given pill than when they do not, a causal relationship is suggested and the pill probably promotes recovery.

When an outcome’s probability without the cause is larger than that with the cause, Δ*p* is negative. This means that there is a causal relationship, but in this case, the cause does not generate the outcome but prevents or inhibits it. For instance, when the probability of recovery is lower when people take a given pill than when they do not take it, there is an inhibitory or preventive causal relationship: the pill is probably preventing recovery.

Most interesting for our present purposes are the cases in which the contingency is null, meaning the causal relationship does not exist. Table 2 shows an example of a fictitious situation in which 80% of the patients who take a given pill recover from a disease, but 80% of patients who do not take it recover just as well. Thus, the outcome is highly probable but does not depend on the presence or absence of the potential cause. In this and other cases, the two probabilities are identical and Δ*p* is therefore 0. In this 0-contingency situation, there is no empirical evidence for assuming that a causal link exists between the potential cause and the effect. Therefore, if a number of experimental participants are shown such a situation and asked to provide a causal judgment, the correct answer should be that there is no causal relation, even though the outcome might occur very frequently and the two events might coincide often (i.e., there might be many cell *a* instances; see Table 2).

**TABLE 2:** **Contingency matrix showing a situation in which the outcome occurs with high probability but with no contingency**.

****	**Outcome present**	**Outcome absent**
Cause present	80	20
Cause absent	80	20

For instance, 80% of the patients who took a pill recovered from a disease, but 80% of patients who did not take the pill recovered just as well.

Experimental participants often overestimate the degree to which the potential cause is actually causing the outcome in null-contingency conditions. This is known as the illusion of causality (or the illusion of control in cases where the potential cause is the behavior of the participant). Even though Δ*p* is generally accepted as a normative index that describes the influence of the cause over the effect, it is now well known that participants do not always base their estimation of causality on Δ*p* (Allan and Jenkins, 1983; Pineño and Miller, 2007). Participants will sometimes use other indexes, and most importantly for our present purposes, their estimations can be biased by other non-normative variables. These variables are the focus of the present report.^1^

In a way, the correct and unbiased detection of whether a causal relationship exists in any situation is equivalent to using a rigorous experimental approach. It requires awareness that human causal inferences are subject to biases, testing what happens when the potential cause is not present, observing a similar number of cases in which the cause is present and absent, being skeptical about mere coincidences, and seeking complete information on all four trial types. It requires knowing when and how to use all these tools. It is important to show people the basic principles of scientific control, as we will see in the experiments studying the illusion of causality described below.

## How to Assess the Illusion

Most of the experiments on how people detect real or illusory causal relationships have used a variation of the same procedure: the contingency judgment task (see special volumes by Shanks et al., 1996; Beckers et al., 2007). This assessment procedure is now relatively standard, which facilitates comparisons across experiments and the evaluation of new applied strategies. This methodology has also been used when there is a need to accurately estimate the degree of illusion of causality that people show before and after receiving training on scientific thinking (Barberia et al., 2013), which is of particular interest for the present report.

In these experiments, participants are exposed to a number of trials in which a given cause is present or absent, followed by the presence or absence of a potential outcome (see Table 1). A typical experiment on the illusion of causality uses a contingency matrix similar to that in Table 2 with a null-contingency situation and manipulation of the different cells to increase or reduce the illusion.

The cover story used in the experiments may be changed to apply to medicine and health (e.g., Matute et al., 2011), stocks and markets (Chapman and Robbins, 1990), foods and allergic reactions (Wasserman et al., 1996), or plants and poisons (Cobos et al., 2007), to name a few examples. In all cases, the aim is to explore how certain manipulations influence the accurate detection of causality. At the end of the experiment, participants are asked to judge the relationship between the potential cause and the potential outcome.

For example, a typical experiment may prompt participants to imagine they are medical doctors. Participants are shown a series of records of fictitious patients suffering from a disease. They see one patient per trial on a computer monitor. Some of these patients take a drug and some do not. Then, some of the patients recover while others do not. In this example, the drug is the potential cause, which might be present or absent in each trial, and the outcome is the recovery from the disease, which might also be present or absent in each trial. The different trial types (*a, b, c, d*) shown in Table 1 are presented in random order. The number of trials (i.e., patients) would usually be between 20 and 100, with 40 to 50 being standard. At the end of the experiment, participants are asked to provide their personal estimation of the relationship between the two events, typically on a scale from 0 (non-effective) to 100 (totally effective).

Among the many possible variations of this task, there is one that deserves special mention. This variable is the active vs. passive role of the participant in this task. In the description of the task so far, participants could passively observe whether the fictitious patients took the drug and then observe whether or not they recovered. This is analogous to vicarious learning by observing or reading about others who have taken the drug. By contrast, in experiments using the active version of the task, participants are shown patients who suffer from the syndrome and are asked, “Would you like to give the drug to this patient?” In these experiments, participants play an active role and decide whether and when the potential cause is presented. After that, the outcome (healing) occurs or does not according to a random or a predetermined sequence that has been pre-programmed by the experimenter. This is an analog of a person who takes a pill to reduce pain. As we will show, some studies have attributed a critical role to this variable, but we argue its effect might sometimes have been confounded with other factors.

In addition to active vs. passive roles of participants, there are many other variants that can be introduced in this task and that have been shown to affect the participants’ estimations of causality. Examples include changing the wording of questions asked at the end of the experiment about the causal relationship (Crocker, 1982; Vadillo et al., 2005, 2011; Collins and Shanks, 2006; De Houwer et al., 2007; Blanco et al., 2010; Shou and Smithson, 2015), the order in which the different trial types are presented (Langer and Roth, 1975; López et al., 1998), the frequency with which judgments are requested (Collins and Shanks, 2002; Matute et al., 2002), the description of the relevant events as causes, predictors, or effects (Waldmann and Holyoak, 1992; Cobos et al., 2002; Pineño et al., 2005), the temporal contiguity between the two events (e.g., Shanks et al., 1989; Wasserman, 1990; Lagnado and Sloman, 2006; Lagnado et al., 2007), and many other variables that fortunately are becoming well known. In the following sections, we will focus on the variables that seem to affect the illusion most critically in cases of null contingency.

## The Probability of the Outcome

One of the first variables found to affect the overestimation of null contingency is the probability of the outcome’s occurrence. Many null-contingency experiments have examined conditions in which a desired outcome occurs by mere chance but with a high probability (e.g., 75% of the occasions, regardless of whether or not the cause is present), which were compared with conditions in which the outcome occurs with low probability (e.g., 25% of the occasions, also independently of the cause’s presence). The illusion of a causal relationship is systematically stronger in the high-outcome conditions than in the low-outcome conditions (Alloy and Abramson, 1979; Allan and Jenkins, 1980, 1983; Matute, 1995; Wasserman et al., 1996; Buehner et al., 2003; Allan et al., 2005; Musca et al., 2010). Thus, when someone is trying to obtain an outcome and the outcome occurs frequently, the feeling that the action is being effective is much stronger than when the outcome occurs rarely. This is usually called outcome-density or outcome-frequency bias.

Knowing that the probability of the outcome affects the perception of causality is important. This knowledge alerts us to conditions that are often more sensitive to causal illusions, such as any disease or pain condition in which spontaneous remissions are frequent. This explains why some life situations are particularly vulnerable to pseudoscientific and magical thinking. For instance, in the USA, alternative medicine is a preferred treatment option for back pain (which has a high percentage of spontaneous remission), with alternative practitioners providing 40% of primary care for back pain (White House Commission on Complementary and Alternative Medicine Policy, 2002). In contrast, alternative medicines are only seldom used to treat disorders where the likelihood of spontaneous remission is low.

Outcome-density bias allows us to predict that null-contingency conditions in which the desired outcome is frequent are susceptible to producing causal illusions. However, it is difficult to prevent those illusions, given that there is little we can do to reduce the probability of the outcome in those cases. In applied settings, the probability of response-independent outcomes occurring is by definition beyond an individual’s control. We can do nothing to change it, so our role should be to raise awareness of the problem and teach people to be vigilant to detect their own illusions in cases where the outcome occurs frequently. A good habit of scientific thinking should therefore be the best defense.

## The Probability of the Cause

The probability of the cause is another variable that has been shown to influence the illusion of causality. With all other things being equal and given the null contingency, the illusion of a cause producing an outcome will be significantly stronger if the cause occurs in 75% of the occasions than when it occurs in 25%. This effect is also called the cause-density or cause-frequency bias and has also been shown in many experiments (Allan and Jenkins, 1983; Wasserman et al., 1996; Perales et al., 2005; Matute et al., 2011; Vadillo et al., 2011; Blanco et al., 2013; Yarritu et al., 2014). The effect is particularly strong when the probability of the outcome is high as well, since there will be more opportunities for coincidences (Blanco et al., 2013).

Fortunately, much can be done to reduce people’s illusions in this case. Even though the probability of the outcome is uncontrollable in real-life situations of null contingency, the probability of the cause is something that can be modified relatively easily. Imagine a popular (and bogus) treatment for a pain condition that has a high outcome probability of spontaneous remissions. As is usually the case in alternative medicine, the treatment involves a very high frequency of the cause (e.g., the pills have to be taken every 2 h). Therefore, we know that this is one of the situations in which the illusion will almost certainly occur. As discussed, it is very difficult to just convince people that their beliefs are false or that their favorite treatment that seems to work so well is innocuous. Importantly, however, we might be able to convince them to reduce the frequency with which they take the pill so that they can at least test what happens when the cause is absent. We know that this strategy works well in the laboratory. If they reduce the cause’s frequency and the outcome persists, then they can realize that the potential cause and outcome are totally independent, and the illusion will be reduced (Blanco et al., 2012; Yarritu et al., 2014). As we will show, this might be done through simple instructions or through any other means that reduce the probability of the potential cause’s occurrence.

Thus, even though it might be difficult to convince people not to use a bogus treatment, we at least know that a good campaign that motivates reduction of treatment frequency can reduce the illusion. Indeed, it has been shown that it is not even necessary that the participants themselves perform the action. In experiments using passive exposure, participants simply observed what happened to fictitious patients in the experiment, as if passively watching television. The results showed that even though participants did not perform the action themselves, observing many vs. few cases in which the cause was present had a significant effect on the illusion that they developed. Those observing fewer patients who followed the treatment reported significantly weaker illusions (e.g., Matute et al., 2011; Vadillo et al., 2011; Yarritu et al., 2014). Thus, there is not only a tendency to use with greater frequency those treatments that seem to be more effective, but also a tendency to perceive more frequently used treatments as more effective. That is, the perception of effectiveness increases usage, but increased usage increases the perception of effectiveness as well (Blanco et al., 2014). Therefore, to show people that a causal relationship does not exist (e.g., that homeopathy does not work), it suffices to ask them to use the potential cause less frequently, or even to show them what happens to people who are not using it (by showing them how people who do not use homeopathy recover just the same, for example).

## Cause-Outcome Coincidences

One of the conditions where most people make systematic errors in estimating causality is when two events keep occurring together in time or in close temporal succession, suggesting that the first one is causing the second one (e.g., Shanks et al., 1989; Wasserman, 1990; Lagnado and Sloman, 2006). We infer causality in those cases, and the inference is often correct, as cause-outcome contiguity is the main cue that humans and other animals can use to infer causal relationships (Wasserman and Neunaber, 1986; Shanks et al., 1989; Wasserman, 1990; Buehner, 2005; Greville and Buehner, 2010). However, when there is no causal relationship and the events still occur in close temporal succession, people will also tend to infer that a causal relationship exists between them, albeit erroneously.

Indeed, of all four trial types in the contingency matrix, people tend to give special weight to trials in cell *a*, for which the cause and the outcome coincide (Jenkins and Ward, 1965; Crocker, 1982; Kao and Wasserman, 1993). This means that even in null-contingency situations, there might be a strong tendency to infer a causal relationship when the number of cause-outcome coincidences (cell *a* trials) is high. In fact, cell *a* is closely related to the factors discussed in the previous two sections, as a high probability of both the cause and the outcome will inevitably produce many cell *a* trials. Not surprisingly, marketing campaigns promoting the use of complementary and alternative medicine or any other miracle products make use of this technique by always informing potential customers about the many successful cases after following their advice, while never mentioning those who succeeded without following their advice. The lesson revealed by laboratory experiments is that informative campaigns from governments and health institutions trying to counteract the advertisement of fraudulent products clearly need to highlight those who did not use the product (i.e., cause-absent trials—cells *c* and *d* in Table 1). It is important to transmit not only truthful but also complete information, including all four trial types (*a, b, c, d*), so that people can make informed decisions.

In sum, the probabilities of both the outcome and the cause are variables that influence the degree of illusion of causality that people will develop in null-contingency conditions. The higher the probabilities of the cause and the outcome, the higher the probability will be that the cause and outcome coincide and the higher the probability of people inferring a causal relation. Moreover, when the probabilities of both the outcome and the cause are high, a special situation occurs in which a majority of the trials involve cell *a* observations. The many coincidences between cause and outcome when both occur frequently seem to give rise to the illusion of causality (Blanco et al., 2013). Thus, to reduce the illusion, we should reduce the probabilities of the outcome and the cause whenever possible and seek for complete information.

**FIGURE 1:**
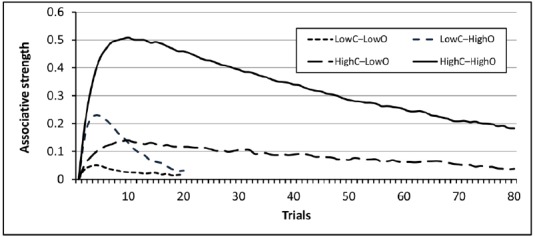
**Results of a computer simulation of the four experimental conditions presented by Blanco et al. (2013, Experiment 1) using the Rescorla–Wagner learning algorithm.** The simulation was conducted using the Java simulator developed by Alonso et al. (2012). For this simulation, the learning rate parameters were set to α_cause_ = 0.3, α_context_ = 0.1, β_outcome_ = β_∼outcome_ = 0.8.

The effects discussed so far do not seem to bias only human judgments. Computer simulations show that the probabilities of the cause and the outcome can also bias machine-learning algorithms designed to detect contingencies. For example, Figure 1 shows the results of a computer simulation based on the popular Rescorla–Wagner learning algorithm (Rescorla and Wagner, 1972). The model tries to associate causes and outcomes co-occurring in an environment while minimizing prediction errors. Each of the four lines shown in Figure 1 denotes the behavior of the model when exposed to each of the four conditions used by Blanco et al. (2013). In this experiment, all participants were exposed to a sequence of trials where the contingency between a potential cause and an outcome was actually 0. The probability of the cause was high (0.80) for half of the participants and low (0.20) for the other half. Orthogonally, the outcome tended to appear with a large probability (0.80) for half of the participants and with low probability (0.20) for the other half.

As shown in Figure 1, the model tends to converge to a low associative strength in all conditions after many trials, a natural result given that the true contingency between cause and outcome is 0 in all cases. However, before the model reaches that asymptote, it is temporarily biased by both the probability of the cause and the probability of the outcome in a way similar to that shown in humans. The model’s ability to learn the 0 contingency is slowest when the probabilities of the cause and outcome are both large. Although the model predicts that the illusion will eventually disappear if a sufficiently large number of trials is provided, the results mimic the causal illusions found in real participants, who also tended to show stronger illusions when both probabilities are large. This has been found in humans using both 50 and 100 training trials (Blanco et al., 2011). This result could also be regarded as a special form of illusory correlation, because both people and the model infer an illusory correlation between the potential cause and the outcome before assuming that the correlation implies a causal relationship.

Interestingly, although there are many other variables that have been described in the literature as influencing the illusion of causality, a close look reveals that many also involve situations with high probability of the outcome, high probability of the cause, or both. Therefore, many of the factors supposedly influencing the illusion might just be instances where these two probabilities are affected. We describe some of these variables below.

## Maximizing the Outcome vs. Testing the Causal Relationship

As explained, some experiments have used a passive version of the contingency learning task, while others have used an active version. In the active version, the participant’s behavior is the potential cause of the outcome: the participant decides when and how often the cause is presented. Therefore, even though the outcome’s occurrence is preprogrammed in the same way in both the active and passive versions, in the active task, participants might increase the number of coincidences between the potential cause and the outcome by chance as a function of when and how often they introduce the cause (Matute, 1996; Hannah and Beneteau, 2009). Thus, the participant could influence the frequencies in the contingency matrix (see Table 1). In these cases, even though the outcome is uncontrollable, the participant may feel as if she were controlling it (Blanco et al., 2011). This is a general feature of instrumental behavior and occurs in real-life uncontrollable situations where the individual is free to introduce the cause. As an example, consider rain dancing. Rain is uncontrollable, but the frequency with which ancient tribes danced was a decision that affected the number of cause-outcome coincidences, which in turn could affect their perception of causality.

This effect depends on the goal and behavior of the participants, so it can also be at least partly modified through instructions to change those goals and the behavior associated with them. Matute (1996) observed that experimental participants showing an illusion of causality were typically exposed to naturalistic-like settings in which their goal was to maximize the outcome, so they tended to act with high probability (of the cause). By contrast, studies reporting accurate detection of null contingencies tended to tell their participants not only that their goal was to assess how much control they had over the outcome (rather than maximizing the outcome), but also how they could best assess the potential relationship. Participants in those experiments were asked to test whether a causal relationship existed and were instructed that the best way to attain that goal was to introduce the cause in about 50% of the trials. This allowed them to learn what happened when the potential cause was present and when it was not (Shanks and Dickinson, 1987; Wasserman, 1990). In a way, the participants were instructed in the principles of scientific control. By prompting them to test the outcomes in the presence and absence of a potential cause, they accurately detected the absence of a contingency. This suggests that accurate detection can be promoted through direct (and rather simple) instructions given to participants.

People tend to act frequently when trying to maximize an outcome, which increases their illusion of causality. Instructing people to reduce the probability of the cause (and refrain from acting on every occasion) shows to be an effective strategy to reduce their illusion (Blanco et al., 2012). This strategy teaches people about what happens when they do or do not act. Not surprisingly, this is equivalent to teaching people the basis of scientific methods and scientific control: to test a causal relationship between two variables, they need to learn to control these variables and see what happens in the presence or absence of the potential cause.

## The Cost of Action—Secondary Effects

We have described a few conditions in which the probability of the cause is explicitly reduced so that people may realize what happens when the cause is absent. As mentioned, this is the basis of the experimental method, which was developed to help scientists perceive causal relationships with accuracy. But in many naturalistic conditions, people tend to act with a very high rate to maximize the outcome, which prevents them from detecting null contingencies. There are many examples. When in pain, we will be more prone to accept any remedy that might be offered. It would be very hard, if not impossible, to ask people to refrain from trying to achieve a desired and truly needed outcome.

There are, however, some factors that by default reduce the probability of action. One is the cost of the action. When an action is expensive (in terms of energy or other costs), people reduce its frequency. Under those conditions, we can predict a reduction in the illusion of causality. One example is provided by the secondary effects of medication. Most drugs produce secondary effects, and taking them has a cost. According to the discussion thus far, this should lead to reduced intake and thus more accurate detection of the null contingency. By the same reasoning, if a drug is not effective but has secondary effects, people should detect its null contingency more easily. However, specious medications that are not effective tend to have neither primary nor secondary effects (as with many complementary medicines such as homeopathy). This might be one of the reasons why many people prefer alternative medicine even when they know that they are not supported by evidence. The presumed lack of side effects makes many people take those medications freely and often, which should lead to a greater illusion that they are effective, as the high probability of the cause is known to increase the illusion.

To test this view, Blanco et al. (2014) adapted the standard contingency learning task described above. Participants were shown records of fictitious patients one by one through a computer screen and decided whether to give these patients a newly discovered drug or to do nothing. They received feedback on whether each patient felt better or not. As in previous experiments, there was no relationship between the drug and healing. The critical manipulation in this experiment was that for one group of participants, patients who took the drug always developed secondary effects, whereas there were no secondary effects for the other group. As expected, the participants for whom the drug produced no secondary effects administered it with greater frequency and were therefore not able to learn that recovery occurred with identical probability regardless of drug administration. Thus, this higher frequency of administration produced stronger causal illusions in the group with no secondary effects. The study demonstrated that not only do we tend to use medicines that we believe to be more effective, but the simple fact of using a medicine frequently promotes the belief that it is effective. This generates a vicious cycle between usage and perception of effectiveness.

These results suggest that one of the paths by which alternative and complementary medicine is becoming so widespread is precisely through their presumed lack of secondary effects. This makes people use these remedies more often than conventional ones (which almost always include secondary effects). Thus, if the outcome occurs with the same probability when not using those remedies, they simply cannot perceive it.

## Depression

Another variable shown to have a profound effect on the perception of null contingencies is mood. A classic study by Alloy and Abramson (1979) showed that people who are depressed are more accurate than non-depressed individuals in detecting their absence of control in non-contingent conditions. This *depressive realism* effect has been replicated under many different conditions (e.g., Moore and Fresco, 2012; Kornbrot et al., 2013; Byrom et al., 2015).

Initially, this effect was explained by assuming that depressed people lack a series of self-serving biases that are assumed to promote well-being in non-depressed people. Non-depressed people would tend to feel that they have control over their environment, which makes them feel well and protects them from depression (Alloy and Abramson, 1988; Taylor and Brown, 1988, 1994; Alloy and Clements, 1992). An alternative view interprets these effects not as a motivational bias, but as the consequence of different cognitive strategies. More specifically, depressed people have been found to differ in how they process cause-absent trials (Msetfi et al., 2005).

Without discussing the merits of these interpretations, we propose another interpretation that is in line with the general framework outlined in this article and which might complement previous proposals on depressive realism. According to Blanco et al. (2009, 2012), one aspect of depression is greater passivity, that is, a reduced ability to initiate voluntary responses. When depressed and non-depressed participants have been compared in experiments on depressive realism, the frequency of occurrence of the cause (the participant’s behavior) has usually not been reported. Thus, non-depressed participants could be acting with greater frequency than depressed participants to obtain the outcome. If this were true, and assuming that the outcome does occur with relatively high frequency in this type of experiment and real-life situations, we can easily anticipate that non-depressed participants will be exposed to a higher number of coincidences (type *a* cells, Table 1). This makes them develop the illusion that they are controlling the outcome. In fact, Blanco et al. (2009, 2012) showed that the depressive realism effect can be due at least in part to the fact that depressed individuals are more passive. They act with less frequency to obtain the outcome and are thus exposed to fewer cause-present trials. As a consequence, their illusion of control is lower.

Therefore, although depressed people seem to be more accurate than non-depressed people in their estimations of null contingency, this does not necessarily mean that they are wiser. Instead, this seems to be an additional consequence of the robust role that the probability of the cause plays in enhancing or reducing the illusions of causality. This highlights the need to teach non-depressed people to be more passive by introducing potential causes in some trials but not in others so that they can learn how much control they actually have over an outcome.

## Personal Involvement

The degree of personal involvement of a participant has been proposed in many earlier reports as one of the most critical variables influencing the results of experiments on illusions of causality and control. In principle, the results of an experiment should differ greatly as a function of whether the participants are personally involved in healing as many fictitious patients as possible, or if they are just observing whether the fictitious patients recover after taking the drug. As mentioned, the illusion of control has been typically interpreted as a form of self-serving bias (Alloy and Abramson, 1988; Taylor and Brown, 1988, 1994; Alloy and Clements, 1992). Therefore, it should not occur when people are just observing causes and effects that have nothing to do with their own behavior.

One of the very few studies that explicitly tested this view was conducted by Alloy et al. (1985). For one group, the potential cause was the participants’ behavior, while the potential cause was a neutral event for the other. The illusion was stronger when the potential cause was the participant’s behavior, which seemed to support the view that personal involvement is necessary to develop the illusion of control. However, a closer look at that experiment reveals that the percentage of trials in which the participants introduced the cause was not reported. This leaves open the possibility that the participants who were personally involved might have been acting with a high probability and thus exposed to a higher probability of the cause than the group of observers. If that was the case, personal involvement might have been confounded with the probability of the cause in previous work.

Thus, we conducted an experiment using a yoked design so that everything remained constant across groups except for personal involvement. One group could administer a drug to fictitious patients, while the other group just observed. The outcome was uncontrollable (preprogrammed) for all participants, but each participant in the yoked group observed just an identical sequence of causes and outcomes generated by a counterpart in the active group. The result was that both groups showed a similar illusion of causality. Participants’ judgments did not differ, regardless of whether they were active or yoked in the experiment (Yarritu et al., 2014). Instead, participants’ judgments were influenced by the probability of the potential cause occurring (recall that the potential cause in this experiment was the administration of the drug, which coincided with the behavior of the participants themselves in the active group, whereas it coincided with the behavior of a third party in the yoked group). Thus, it seemed that the probability of the cause was a stronger determinant than personal involvement in the development of the illusion.

To confirm this finding, Yarritu et al. (2014) conducted another experiment in which they explicitly manipulated the probability of the potential cause and the degree of personal involvement. Half of the participants were asked to try to heal as many patients as possible, while the other half just observed what their partners were doing. To increase the motivation of the active participants, they were made aware that their performance was being monitored by peer participants observing the screen from a cloned one in an adjacent cubicle. Orthogonally, half of the participants in each group were given a short supply of the drug so that they were forced to administer it with low probability to just a few patients. The other half was given a large supply of the drug and induced to administer the drug with high frequency. The results confirmed our previous findings that the probability with which the drug was given had a stronger effect on judgments than being personally involved in trying to heal the patients (vs. just observing).

This is not to say that all cases showing that personal involvement increases the illusion of control can be reduced to a probability of the cause effect. Quite possibly, however, some confusion has existed in the literature because people who are personally involved tend to act more often to obtain the desired outcome than those who are not involved. The probability of introducing the potential cause is higher in those involved, and as previously shown, this can increase the number of coincidences and therefore the illusion as well. As a consequence, it is necessary to distance oneself in critical life conditions in which we are personally involved or to let more objective and neutral observers help us judge whether a causal relationship exists, because our intuitions will surely be wrong.

## When There are Several Potential Causes

There are times when the occurrence of coincidences is not enough to strengthen the perception of causality. When multiple possible causes are presented together and followed by an outcome, causes tend to compete among themselves for association with the outcome. Even when there might be many cause-outcome coincidences, they may not be effective in strengthening the attribution of causality to one of these causes if the other cause is more intense or had a previous association with the outcome (Rescorla and Wagner, 1972). These effects of competition between several potential causes are well known in both humans and other animals (e.g., Shanks, 2007; Wheeler and Miller, 2008).

Taking this idea a bit further, we could also predict that in situations in which the outcome is independent of behavior, informing people about potential alternative explanations for the outcome should also reduce their illusion of causality. This idea was tested by Vadillo et al. (2013), who instructed one of their groups about a potential alternative explanation for the outcome. As expected, the group with an alternative explanation to which the outcome could easily be attributed showed a reduced illusion of causality compared to the group that received no suggestions about alternative explanations. Thus, informing people about the existence of alternative causes can reduce the illusion.

However, the availability of alternative causes is not always beneficial. There are cases in which people might choose the wrong cause if two are available. As an example, consider a patient taking placebo pills for better sleep. A friend tells him that the pills are just sugar and that he should start a new treatment. He hesitates but finally decides to follow his friend’s advice. He visits a psychologist and starts following an evidence-based treatment. However, just in case, he does not quit the placebo pills and keeps using them as complementary therapy. Doing so implies that it will be impossible for him to tell whether any improvement is due to the new treatment or to the placebo pills. Even if the new treatment works, its effect can be wrongly attributed to the old placebo pills.

Alternative therapies are often used in addition to other therapies under the belief that they cannot be harmful if used in this way. Indeed, many people would agree that alternative therapy might be harmful because it might make people not follow effective treatments. But when used as a complement, the alternative seems to be absolutely harmless and almost no one would oppose this use. However, as we have been advancing, the availability of more than one potential cause can result in a competition between both causes, so that if one is considered to be a strong candidate, the other will be seen as a weak one. Indeed, many experiments with both humans and other animals have reported that when two causes are presented together and followed by an outcome, one of the causes having a previous history of association with that outcome can compete with the attribution of causal strength to the most recent cause (Shanks and Dickinson, 1987; Shanks, 2007; Wheeler and Miller, 2008; Boddez et al., 2014).

With this in mind, Yarritu et al. (2015) asked whether the same effect would occur when the previous history of one of the causes with the outcome was just illusory. They asked two groups to assess the effectiveness of drug A in the task described in previous sections. During Phase 1, the percentage of trials in which the fictitious patients recovered was the same regardless of whether they took the drug and was determined by a preprogrammed random sequence. However, a strong illusion that the drug was effective was induced in one group, while a weak illusion was induced in the other group. This was done by manipulating the probability of the cause (the fictitious patients taking the drug). Then, in Phase 2, all participants were exposed to a series of patients who either took a combined treatment of drug A with a new drug B or received no treatment, after which the patients recovered or did not. The percentage of trials in which the patients recovered after taking the combined medication was higher than in trials with recovery without medication, and these percentages were identical for all participants. That is, during Phase 2 drug B was effective. However, when asked about the effectiveness of drug B in a subsequent test phase, the judgments were lower for the group that had developed the strong illusion about drug A in Phase 1. The previous illusion that medicine A was effective reduced the ability to detect the effectiveness of medicine B during Phase 2. This suggests that specious medication, even when complementary, can produce more harm than people usually expect.

## Aversive Conditions: Just the Other Way Around?

Throughout this article, we have been assuming that the illusion of causality occurs in appetitive conditions when people are trying to obtain a desired event. However, many superstitions and illusions of causality take place in aversive conditions in which the outcome is an undesired one such as bad luck or misfortune (e.g., Blum and Blum, 1974; Aeschleman et al., 2003; Bloom et al., 2007; Zhang et al., 2014). We should distinguish at least two types of aversive or negative conditions in which the null contingency may take place. The first one parallels escape and avoidance behavior. Even though there is no contingency between cause and outcome, it will look as if the participants’ behavior is followed by the *termination (or avoidance)* of an aversive event. A common example is touching wood to avoid misfortune. This first type of negative superstition works in much the same way as the one with the appetitive outcomes discussed thus far. People will typically perform actions with high probability in their attempt to escape or avoid an aversive stimulus as often as possible (Matute, 1995, 1996; Blanco and Matute, 2015). Therefore, most of the strategies suggested to reduce the illusion of causality should also be useful in these cases.

The second type parallels a punishment condition. The participant’s behavior does not produce bad luck, but it seems *as if* this were the case. For instance, sitting in row number 13 or seeing a black cat is considered to bring bad luck. These cases work differently from the ones described. When aversive consequences follow an action (or seem to follow them), people tend to reduce their behavior (i.e., the probability of the cause). This is important for understanding the difference between these punishment-like conditions and the appetitive ones. People tend to act with less frequency in this case, in contrast to appetitive conditions. Thus, to help them realize that the outcome is independent of their behavior, our strategy should be opposite to that used in appetitive conditions.

To test this view, Matute and Blanco (2014) used the same instructions shown to reduce illusions in appetitive conditions in punishment-like conditions. The result was that asking people to reduce the probability of the cause (i.e., their actions) while warning them about potential alternative causes of the outcome, increased, rather than reduced, the illusion. The reason is probably that when aversive uncontrollable outcomes follow behavior and people act frequently, as they do by default, their behavior seems to be punished, so they feel they are not controlling the outcome. However, when a potential alternative cause for those aversive outcomes is available and people reduce the frequency with which they act, and even so the aversive outcomes keep occurring, it becomes clearer for people that they are not responsible for the occurrence of the outcomes. They no longer feel that their behavior is being punished and thus their illusion of control increases. Therefore, rather than instructing people to reduce the number of trials in which the cause is present, the best strategy in punishment-like conditions is asking people to increase cause-present trials. In that way, it will be easier for them to detect that they have no control. As an example, rather than asking people to refrain from selecting row number 13th we should ask them to select row number 13th with greater frequency so that their negative illusion can be reduced.

## Developing an Educational Strategy

There has been a long debate in scientific education on whether students learn better through personal discovery or through more traditional instruction, but recent reports are showing that to learn about scientific methods, direct instruction is preferred (e.g., Klahr and Nigam, 2004). Thus, the advantages of direct instruction could be used to prevent illusions of causality. However, one serious potential problem is that people may ignore recommendations because they are not motivated based on beliefs that they are free from biases. Many people can recognize cognitive biases in other people but are terrible at recognizing their own biases (Pronin et al., 2004, 2002). We can hypothesize that making people realize their perception of causality is far from perfect will motivate them to learn about scientific methods that would help them assess causality accurately.

Following this idea, Barberia et al. (2013) told a group of adolescents that they had developed a miracle product that would help them improve their physical and cognitive abilities. This was conducted quite theatrically, and the teenagers were allowed to try the properties of the product (a piece of regular ferrite) through several cognitive and physical exercises. Once the adolescents were made to believe that the bogus product was really effective, they were ready to listen to possible ways in which they could have assessed the efficacy of the ferrite more accurately. They received a seminar on scientific methods emphasizing the importance of controlling for extraneous or confounding variables. They were also shown what the product really was and how they had been victims of their own biases. Finally, their illusion of causality was assessed using the standard procedure described in previous sections.

The result was that the students that had undergone the intervention (including both the experience with the bogus product and the tutorial on experimental methods) reduced their probability of introducing the cause (their probability of acting) and developed a significantly weaker causal illusion in the standardized judgmental task compared to a control group of naïve participants who did not receive the intervention. We cannot be sure of the key aspect in the success of the procedure, but we conjecture that making the students aware of their own biases might be critical in enhancing the impact that the more academic explanation of scientific methods had over their behavior and thinking. Future interventions should focus on disentangling the key components that made the intervention successful, such as the role of the initial phase. The results suggest that contrary to the extended view that cognitive biases cannot be counteracted, there are procedures that work and need to be documented, as they can help teach people how to think more scientifically and reduce their causal illusions.

We are not aware of many other systematic studies on reducing the illusion of causality or even attempts to reduce other biases (e.g., Arkes, 1991; Larrick, 2004; Schmaltz and Lilienfeld, 2014). As Lilienfeld et al. (2009) state, it is strange that with so much research being conducted on cognitive biases in the last decades that so little has been done in relation to debiasing. We hope that we have contributed to developing evidence-based strategies that can improve the teaching of scientific methods and reduce the illusion of causality.

## Discussion

Several recommendations on how to reduce the illusion of causality can be extracted from the experiments reported. First, we know that the illusion of cause and effect will be weaker when the desired outcome rarely occurs. Although this variable is usually beyond our control in null-contingency conditions, it provides an important cue to anticipate the conditions under which it is more likely to observe superstitions and causal illusions.

The illusion will also be weaker when the probability of the cause is low. This is a variable that can be controlled and has shown to affect the illusion of causality in many experiments in many different laboratories (Allan and Jenkins, 1983; Wasserman et al., 1996; Perales et al., 2005; Vadillo et al., 2011). Therefore, it can be used to reduce the illusion. Specious product advertisements mention only the cases in which the cause is present (product use) and the product works effectively (cell *a* instances in Table 1). Thus, one very simple strategy that governments could use is to make sure that the data from those cases without the potential cause are also presented during those marketing campaigns. A related and even better strategy that governments should use is teaching people how to use the data. Governments could teach people to realize that they need to ask for information about cause-absent cases when such information is not readily available. In other words, governments could teach people (not just science students) how to make better use of scientific thinking in their everyday life.

The experiments also showed that the effect of the probability of the cause (cause-density bias) is also observed indirectly when someone is depressed because they are more passive. By extension, this effect is also observed under any other conditions that might make people reduce their default tendency to introduce a cause in almost any occasion. Other examples are cases in which the potential cause is costly or has undesired collateral effects. People do not introduce the cause often in those cases and thus can learn what happens when they do nothing. Likewise, the illusion is also weaker when people are just observing the cause and the effect co-occur than when their own behavior is the potential cause. Moreover, the illusion is stronger when people are trying to obtain an outcome, such as when a doctor in an emergency room is trying to help patients. This contrasts with cases of people trying to find their degree of causality, such as when a scientist is testing the effect of a drug on a health problem.

In addition, there are cases in which the results are reversed, such as when the cause is followed by an undesired event, as in punishment-like conditions. There are also cases in which the principles of cue competition apply because there is more than one potential cause, with some causes preventing attribution to other causes simultaneously occurring. These cue competition effects reduce the illusion in some cases and enhance it in others. This means that there are many variables that are currently very well known, have been tested in many experiments, and have predictable effects. Thus, we can now anticipate the cases that will produce a stronger or a weaker illusion in most people. This knowledge could be used to make people more alert when illusions are most probable. We also know from the experiments that in all cases, the illusion can be reduced if people are taught to reduce the probability of introducing the potential cause by learning the basic principles of scientific control and the experimental paradigm.

With this in mind, we outlined an educational strategy as an example of what could be done to provide adolescents with a tool to become less vulnerable to the illusion of causality. The basic idea is not new. It should consist of teaching how to think scientifically and apply basic scientific principles to areas of their own interest. The procedure includes a strategy to motivate teenagers to learn how to protect themselves from their own biases. This is a critical part of teaching scientific thinking. Many people have no interest in learning about scientific thinking because they are not aware that they need to correct their own biases in their everyday life. However, once people are shown how fallible their intuitions are, they should be willing to learn about tools to prevent errors. One way to motivate people is by defeating them before offering the tutorial (Barberia et al., 2013). Another is by showing them examples of common superstitions, pseudosciences, and myths that they might believe so that they can learn what their error is and how to use scientific thinking to overcome the error (Schmaltz and Lilienfeld, 2014).

We have reduced most conditions leading to the attenuation of the illusion of causality in the literature to cases in which the cause was presented with a probability closer to 50%. Controlling the probability of the potential cause is a very important factor in reducing the illusion, which has been shown in many different experiments. Indeed, this approach is at the heart of the experimental method. If we look at how scientists manipulate the probability of a cause when they perform an experiment, they might avoid having 50 participants in one group and 5 in another, or they might try to give cause-present and cause-absent groups a similar weight and size. However, this is obviously not the only factor. Reduction in cognitive biases and ungrounded beliefs has been demonstrated by encouraging a more analytical and distant style of thinking, as opposed to the default of an intuitive, fast, and more emotional way of thinking in situations that are not necessarily contaminated with a high probability of the potential cause (Frederick, 2005; Kahneman, 2011; Stanovich, 2011; Ziegler and Tunney, 2012; Evans and Stanovich, 2013). This includes a reduction of religious and teleological beliefs (Gervais and Norenzayan, 2012; Kelemen et al., 2013), as well as a reduction of several cognitive biases such as confirmation biases (Galinsky and Moskowitz, 2000; Galinsky and Ku, 2004) and framing (Keysar et al., 2012; Costa et al., 2014). Our preliminary research suggests that this approach based on the literature on general cognitive biases can also help reduce the illusion of causality (Díaz-Lago and Matute, 2014).

As Lilienfeld et al. (2009) have noted, designing a worldwide strategy to reduce cognitive biases would be the greatest contribution that psychology could make to humanity, as it would eliminate so much suffering and intolerance. We have reviewed some of the evidence about two of these biases—the illusion of causality and the illusion of control—and how they can be reduced. We hope that this will contribute to increasing awareness of these biases and of ways to effectively reduce them in real life. Of course, this is not to say that analytical thinking should always be preferred over fast intuition. As many have already noted, there are times when intuitive judgments are more accurate than analytical ones (Kruglanski and Gigerenzer, 2011; Phua and Tan, 2013; Evans, 2014). Thus, the aim when teaching scientific methods should not only be mastering the ability to think scientifically, but perhaps most importantly, the ability to detect when this mode of thinking should be used.

### Conflict of Interest Statement

The authors declare that the research was conducted in the absence of any commercial or financial relationships that could be construed as a potential conflict of interest.

## Footnotes

1 Note that all the situations described hereafter involve neither correlation nor causation. They should not be confounded with situations leading to the well-known *cum hoc ergo propter hoc* bias, in which people assume that there is causation when only a correlation exists.

## References

[B1] Achenbach J. (2015). Why Do Many Reasonable People Doubt Science? National Geographic. Available at: http://ngm.nationalgeographic.com/ [accessed March 28, 2015].

[B2] Aeschleman S. R. Rosen C. C. Williams M. R. (2003). The effect of non-contingent negative and positive reinforcement operations on the acquisition of superstitious behaviors. Behav. Processes 61, 37–45. 10.1016/S0376-6357(02)00158-4 12543481

[B3] Allan L. G. (1980). A note on measurement of contingency between two binary variables in judgement tasks. Bull. Psychon. Soc. 15, 147–149.

[B4] Allan L. G. (1993). Human contingency judgments: rule based or associative? Psychol. Bull. 114, 435–448. 8272465 10.1037/0033-2909.114.3.435

[B5] Allan L. G. Jenkins H. M. (1980). The judgment of contingency and the nature of the response alternatives. Can. J. Exp. Psychol. 34, 1–11. 10.1037/h0081013

[B6] Allan L. G. Jenkins H. M. (1983). The effect of representations of binary variables on judgment of influence. Learn. Motiv. 14, 381–405. 10.1016/0023-9690(83)90024-3

[B7] Allan L. G. Siegel S. Tangen J. M. (2005). A signal detection analysis of contingency data. Learn. Behav. 33, 250–263. 10.3758/BF03196067 16075843

[B8] Alloy L. B. Abramson L. Y. (1979). Judgment of contingency in depressed and nondepressed students: sadder but wiser? J. Exp. Psychol. Gen. 108, 441–485. 10.1037/0096-3445.108.4.441 528910

[B9] Alloy L. B. Abramson L. Y. (1988). “Depressive realism: four theoretical perspectives,” in Cognitive Processes in Depression, ed. Alloy L. B. (New York, NY: Guilford University Press), 223–265.

[B10] Alloy L. B. Abramson L. Y. Kossman D. A. (1985). “The judgment of predictability in depressed and nondepressed college students,” in Affect, Conditioning, and Cognition: Essays on the Determinants of Behavior, eds Brush F. R. Overmier J. B. (Hillsdale, NJ: Lawrence Erlbaum), 229–246.

[B11] Alloy L. B. Clements C. M. (1992). Illusion of control: invulnerability to negative affect and depressive symptoms after laboratory and natural stressors. J. Abnorm. Psychol. 101, 234–245. 1583214 10.1037//0021-843x.101.2.234

[B12] Alonso E. Mondragón E. Fernández A. (2012). A Java simulator of Rescorla and Wagner’s prediction error model and configural cue extensions. Comput. Methods Programs 108, 346–355. 10.1016/j.cmpb.2012.02.004 22420931

[B13] Arkes H. R. (1991). Costs and benefits of judgment errors: implications for debiasing. Psychol. Bull. 110, 486–498. 10.1037//0033-2909.110.3.486

[B14] Barberia I. Blanco F. Cubillas C. P. Matute H. (2013). Implementation and assessment of an intervention to debias adolescents against causal illusions. PLoS ONE 8:e71303. 10.1371/journal.pone.0071303 23967189 PMC3743900

[B15] Beckers T. De Houwer J. Matute H. (2007). Human Contingency Learning: Recent Trends in Research and Theory. A Special Issue of The Quarterly Journal of Experimental Psychology. Hove: Psychology Press.

[B16] Blanco F. Barberia I. Matute H. (2014). The lack of side effects of an ineffective treatment facilitates the development of a belief in its effectiveness. PLoS ONE 9:e84084. 10.1371/journal.pone.0084084 24416194 PMC3885525

[B17] Blanco F. Matute H. (2015). Exploring the factors that encourage the illusions of control: the case of preventive illusions. Exp. Psychol. 62, 131–142. 10.1027/1618-3169/a000280 25384640 PMC4614377

[B18] Blanco F. Matute H. Vadillo M. A. (2010). Contingency is used to prepare for outcomes: implications for a functional analysis of learning. Psychon. Bull. Rev. 17, 117–121. 10.3758/PBR.17.1.117 20081171

[B19] Blanco F. Matute H. Vadillo M. A. (2011). Making the uncontrollable seem controllable: the role of action in the illusion of control. Q. J. Exp. Psychol. 64, 1290–1304. 10.1080/17470218.2011.552727 21432736

[B20] Blanco F. Matute H. Vadillo M. A. (2012). Mediating role of activity level in the depressive realism effect. PLoS ONE 7:e46203. 10.1371/journal.pone.0046203 23029435 PMC3459889

[B21] Blanco F. Matute H. Vadillo M. A. (2013). Interactive effects of the probability of the cue and the probability of the outcome on the overestimation of null contingency. Learn. Behav. 41, 333–340. 10.3758/s13420-013-0108-8 23529636

[B22] Blanco H. Matute H. Vadillo M. A. (2009). Depressive realism: wiser or quieter? Psychol. Rec. 59, 551–562.

[B23] Bloom C. M. Venard J. Harden M. Seetharaman S. (2007). Non-contingent positive and negative reinforcement schedules of superstitious behaviors. Behav. Process. 75, 8–13. 10.1016/j.beproc.2007.02.010 17353100

[B24] Bloom P. Weisberg D. S. (2007). Childhood origins of adult resistance to science. Science 316, 996–997. 10.1126/science.1133398 17510356

[B25] Blum S. H. Blum L. H. (1974). Do’s and dont’s: an informal study of some prevailing superstitions. Psychol. Rep. 35, 567–571. 10.2466/pr0.1974.35.1.567

[B26] Boddez Y. Haesen K. Baeyens F. Beckers T. (2014). Selectivity in associative learning: a cognitive stage framework for blocking and cue competition phenomena. Front. Psychol. 5:1305. 10.3389/fpsyg.2014.01305 25429280 PMC4228836

[B27] Buehner M. J. (2005). Contiguity and covariation in human causal inference. Learn. Behav. 33, 230–238. 10.3758/BF03196065 16075841

[B28] Buehner M. J. Cheng P. W. Clifford D. (2003). From covariation to causation: a test of the assumption of causal power. J. Exp. Psychol. Learn. Mem. Cogn. 29, 1119–1140. 10.1037/0278-7393.29.6.1119 14622051

[B29] Byrom N. C. Msetfi R, M. Murphy R. A. (2015). Two pathways to causal control: use and availability of information in the environment in people with and without signs of depression. Acta Psychol. 157, 1–12. 10.1016/j.actpsy.2015.02.004 25703605

[B30] Carroll R. (2015). Too Rich to Get Sick? Disneyland Measles Outbreak Reflects Anti-vaccination Trend. The Guardian. Available at: http://www.theguardian.com [accessed February 6, 2015].

[B31] Chapman G. B. Robbins S. J. (1990). Cue interaction in human contingency judgment. Mem. Cogn. 18, 537–545. 2233266 10.3758/bf03198486

[B32] Cheng P. W. Novick L. R. (1992). Covariation in natural causal induction. Psychol. Rev. 99, 365–382. 10.1037/0033-295X.99.2.365 1594730

[B33] Cobos P. L. López F. J. Caño A. Almaraz J. Shanks D. R. (2002). Mechanisms of predictive and diagnostic causal induction. J. Exp. Psychol. Anim. Behav. Process. 28, 331–346. 10.1037/0097-7403.28.4.331 12395491

[B34] Cobos P. L. López F. J. Luque D. (2007). Interference between cues of the same outcome depends on the causal interpretation of the events. Q. J. Exp. Psychol. 60, 369–386. 10.1080/17470210601000961 17366306

[B35] Collins D. J. Shanks D. R. (2002). Momentary and integrative response strategies in causal judgment. Mem. Cogn. 30, 1138–1147. 10.3758/BF03194331 12507378

[B36] Collins D. J. Shanks D. R. (2006). Conformity to the power PC theory of causal induction depends on the type of probe question. Q. J. Exp. Psychol. 59, 225–232. 10.1080/17470210500370457 16618631

[B37] Costa A. Foucart A. Arnon I. Aparici M. Apesteguia J. (2014). “Piensa” twice: on the foreign language effect in decision making. Cognition 130, 236–254. 10.1016/j.cognition.2013.11.010 24334107

[B38] Crocker J. (1981). Judgment of covariation by social perceivers. Psychol. Bull. 90, 272–292. 10.1037//0033-2909.90.2.272

[B39] Crocker J. (1982). Biased questions in judgment of covariation studies. Pers. Soc. Psychol. Bull. 8, 214–220. 10.1177/0146167282082005

[B40] De Houwer J. Vandorpe S. Beckers T. (2007). Statistical contingency has a different impact on preparation judgements than on causal judgements. Q. J. Exp. Psychol. 60, 418–432. 10.1080/17470210601001084 17366309

[B41] Díaz-Lago M. Matute H. (2014). Foreign language reduces the illusion of causality. *Paper Presented at the Joint Meeting of the Sociedad Española de Psicología Experimental (SEPEX) and Sociedad Española de Psicofisiología y Neurociencia Cognitiva y Afectiva (SEPNECA)*. Murcia, Spain. 10.1177/1747021818755326 PMC6295649 29451106

[B42] Eiser J. R. Stafford T. Henneberry J. Catney P. (2009). “Trust me, I’m a scientist (not a developer)”: perceived expertise and motives as predictors of trust in assessment of risk from contaminated land. Risk Anal. 29, 288–297. 10.1111/j.1539-6924.2008.01131.x 18826417

[B43] Ernst E. (2015). A Scientist in Wonderland: A Memoir of Searching for Truth and Finding Trouble. Exeter: Imprint Academic.

[B44] European Commission. (2005). Special Eurobarometer 224: Europeans, Science and Technology. EBS Report No. 224. Brussels: European Commission.

[B45] European Commission. (2010). Special Eurobarometer 340: Science and Technology. EBS Report No. 340. Brussels: European Commission.

[B46] Evans J. S. B. T. (2014). Two minds rationality. Think. Reason. 20, 129–146. 10.1080/13546783.2013.845605

[B47] Evans J. S. B. T. Stanovich K. E. (2013). Dual-process theories of higher cognition: advancing the debate. Perspect. Psychol. Sci. 8, 223–241. 10.1177/1745691612460685 26172965

[B48] Frederick S. (2005). Cognitive reflection and decision making. J. Econ. Perspect. 19, 25–42. 10.1257/089533005775196732

[B49] Freckelton I. (2012). Death by homeopathy: issues for civil, criminal and coronial law and for health service policy. J. Law Med. 19, 454–478. 22558899

[B50] Galinsky A. D. Ku G. (2004). The effects of perspective-taking on prejudice: the moderating role of self-evaluation. Personal. Soc. Psychol. Bull. 30, 594–604. 10.1177/0146167203262802 15107159

[B51] Galinsky A. Moskowitz G. (2000). Perspective-taking: decreasing stereotype expression, stereotype accessibility, and in-group favoritism. J. Pers. Soc. Psychol. 78, 708–724. 10.1037/0022-3514.78.4.708 10794375

[B52] Gervais W. M. Norenzayan A. (2012). Analytic thinking promotes religious disbelief. Science 336, 493–496. 10.1126/science.1215647 22539725

[B53] Gough D. Tripney J. Kenny C. Buk-Berge E. (2011). Evidence Informed Policymaking in Education in Europe: EIPEE Final Project Report. London: Institute of Education, University of London.

[B54] Greville W. J. Buehner M. J. (2010). Temporal predictability facilitates causal learning. J. Exp. Psychol. Gen. 139, 756–771. 10.1037/a0020976 21038987

[B55] Haberman C. (2015). A Discredited Vaccine Study’s Continuing Impact on Public Health. The New York Times. Available at: http://www.nytimes.com [accessed February 6, 2015].

[B56] Hamilton D. L. Gifford R. K. (1976). Illusory correlation in interpersonal perception: a cognitive basis of stereotypic judgments. J. Exp. Soc. Psychol. 12, 392–407. 10.1016/S0022-1031(76)80006-6

[B57] Hannah S. D. Beneteau J. L. (2009). Just tell me what to do: bringing back experimenter control in active contingency tasks with the command-performance procedure and finding cue density effects along the way. Can. J. Exp. Psychol. 63, 59–73. 10.1037/a0013403 19271817

[B58] House of Commons Science and Technology Committee. (2010). Evidence Check 2: Homeopathy. HCSTC Report No. 4 of Session 2009–2010. London: The Stationery Office.

[B59] Hutson M. (2015). The Science of Superstition. The Atlantic. Available at: http://www.theatlantic.com/ [accessed February 16, 2015].

[B60] Jenkins H. M. Ward W. C. (1965). Judgment of contingency between responses and outcomes. Psychol. Monogr. 79, 1–17. 10.1037/h0093874 14300511

[B61] Johnson D. D. P. (2004). Overconfidence and War: The Havoc and Glory of Positive Illusions. Cambridge, MA: Harvard University Press.

[B62] Kahneman D. (2011). Thinking, Fast and Slow. New York: Farrar, Straus and Giroux.

[B63] Kao S.-F. Wasserman E. A. (1993). Assessment of an information integration account of contingency judgment with examination of subjective cell importance and method of information presentation. J. Exp. Psychol. Learn. Mem. Cogn. 19, 1363–1386. 10.1037/0278-7393.19.6.1363

[B64] Kelemen D. Rottman J. Seston R. (2013). Professional physical scientists display tenacious teleological tendencies: purpose-based reasoning as a cognitive default. J. Exp. Psychol. Gen. 142, 1074–1083. 10.1037/a0030399 23067062

[B65] Keysar B. Hayakawa S. An S. G. (2012). The foreign language effect: thinking in a foreign tongue reduces decision biases. Psychol. Sci. 23, 661–668. 10.1177/0956797611432178 22517192

[B66] Klahr D. Nigam M. (2004). The equivalence of learning paths in early science instruction: effects of direct instruction and discovery learning. Psychol. Sci. 15, 661–667. 10.1111/j.0956-7976.2004.00737.x 15447636

[B67] Kornbrot D. E. Msetfi R. M. Grimwood M. J. (2013). Time perception and depressive realism: judgment type, psychophysical functions and bias. PLoS ONE 8:e71585. 10.1371/journal.pone.0071585 23990960 PMC3749223

[B68] Kruglanski A. W. Gigerenzer G. (2011). Intuitive and deliberative judgements are based on common principles. Psychol. Rev. 118, 97–109. 10.1037/a0020762 21244188

[B69] Lagnado D. A. Sloman S. A. (2006). Time as a guide to cause. J. Exp. Psychol. Learn. Mem. Cogn. 32, 451–460. 10.1037/0278-7393.32.3.451 16719658

[B70] Lagnado D. A. Waldmann M. R. Hagmayer Y. Sloman S. A. (2007). “Beyond covariation: cues to causal structure,” in Causal Learning: Psychology, Philosophy, and Computation, eds Gopnik A. Schultz L. (New York: Oxford University Press), 154–172.

[B71] Langer E. J. Roth J. (1975). Heads I win, tails it’s chance: the illusion of control as a function of the sequence of outcomes in a purely chance task. J. Pers. Soc. Psychol. 32, 951–955. 10.1037/0022-3514.32.6.951

[B72] Larrick R. P. (2004). “Debiasing,” in Blackwell Handbook of Judgment and Decision Making, eds Koehler D. J. Harvey N. (Oxford: Blackwell), 316–338.

[B73] Lewandowsky S. Ecker U. K. H. Seifert C. M. Schwarz N. Cook J. (2012). Misinformation and its correction continued influence and successful debiasing. Psychol. Sci. Public Interest 13, 106–131. 10.1177/1529100612451018 26173286

[B74] Lilienfeld S. O. Ammirati R. David M. (2012). Distinguishing science from pseudoscience in school psychology: science and scientific thinking as safeguards against human error. J. Sch. Psychol. 50, 7–36. 10.1016/j.jsp.2011.09.006 22386075

[B75] Lilienfeld S. O. Ammirati R. Landfield K. (2009). Giving debiasing away. Can psychological research on correcting cognitive errors promote human welfare? Perspect. Psychol. Sci. 4, 390–398. 10.1111/j.1745-6924.2009.01144.x 26158987

[B76] Lilienfeld S. O. Ritschel L. A. Lynn S. J. Cautin R. L. Latzman R. D. (2014). Why ineffective psychotherapies appear to work: a taxonomy of causes of spurious therapeutic effectiveness. Perspect. Psychol. Sci. 9, 355–387. 10.1177/1745691614535216 26173271

[B77] Lindeman M. Svedholm A. M. (2012). What’s in a term? Paranormal, superstitious, magical and supernatural beliefs by any other name would mean the same. Rev. Gen. Psychol. 16, 241–255. 10.1037/a0027158

[B78] López F. J. Shanks D. R. Almaraz J. Fernández P. (1998). Effects of trial order on contingency judgments: a comparison of associative and probabilistic contrast accounts. J. Exp. Psychol. Learn. Mem. Cogn. 24, 672–694. 10.1037/0278-7393.24.3.672

[B79] Malmendier U. Tate G. A. (2005). CEO overconfidence and corporate investment. J. Finance 60, 2661–2700. 10.1111/j.1540-6261.2005.00813.x

[B80] Matute H. (1995). Human reactions to uncontrollable outcomes: further evidence for superstitions rather than helplessness. Q. J. Exp. Psychol. 48, 142–157.

[B81] Matute H. (1996). Illusion of control: detecting response-outcome independence in analytic but not in naturalistic conditions. Psychol. Sci. 7, 289–293. 10.1111/j.1467-9280.1996.tb00376.x

[B82] Matute H. Blanco F. (2014). Reducing the illusion of control when the action is followed by undesired outcomes. Psychon. Bull. Rev. 21, 1087–1093. 10.3758/s13423-014-0584-7 24448764

[B83] Matute H. Vegas S. De Marez P. J. (2002). Flexible use of recent information in causal and predictive judgments. J. Exp. Psychol. Learn. Mem. Cogn. 28, 714–725. 10.1037/0278-7393.28.4.714 12109763

[B84] Matute H. Yarritu I. Vadillo M. A. (2011). Illusions of causality at the heart of pseudoscience. Br. J. Psychol. 102, 392–405. 10.1348/000712610X532210 21751996

[B85] Moore D. W. (2005). Three in Four Americans Believe in Paranormal. Princeton: Gallup News Service.

[B86] Moore M. T. Fresco D. M. (2012). Depressive realism: a meta-analytic review. Clin. Psychol. Rev. 32, 496–509. 10.1016/j.cpr.2012.05.004 22717337

[B87] Msetfi R. M. Murphy R. A. Simpson J. (2007). Depressive realism and the effect of intertrial interval on judgements of zero, positive, and negative contingencies. Q. J. Exp. Psychol. 60, 461–481. 10.1080/17470210601002595 17366312

[B88] Msetfi R. M. Murphy R. A. Simpson J. Kornbrot D. E. (2005). Depressive realism and outcome density bias in contingency judgments: the effect of the context and inter-trial interval. J. Exp. Psychol. Gen. 134, 10–22. 10.1037/0096-3445.134.1.10 15702960

[B89] Murphy R. A. Schmeer S. Vallée-Tourangeau F. Mondragón E. Hilton D. (2011). Making the illusory correlation effect appear and then disappear: the effects of increased learning. Q. J. Exp. Psychol. 64, 24–40. 10.1080/17470218.2010.493615 20623441

[B90] Musca S. C. Vadillo M. A. Blanco F. Matute H. (2010). The role of cue information in the outcome-density effect: evidence from neural network simulations and a causal learning experiment. Conn. Sci. 20, 177–192. 10.1080/09540091003623797

[B91] National Health and Medical Research Council. (2015). NHMRC Statement on Homeopathy and NHMRC Information Paper: Evidence on the Effectiveness of Homeopathy for Treating Health Conditions (NHMRC Publication No. CAM02). Available at: https://www.nhmrc.gov.au/guidelines-publications/cam02 [accessed March 28, 2015].

[B92] Nyhan B. Reifler J. (2015). Does correcting myths about the flu vaccine work? An experimental evaluation of the effects of corrective information. Vaccine 33, 459–464. 10.1016/j.vaccine.2014.11.017 25499651

[B93] Perales J. C. Catena A. Shanks D. R. González J. A. (2005). Dissociation between judgments and outcome-expectancy measures in covariation learning: a signal detection theory approach. J. Exp. Psychol. Learn. Mem. Cogn. 31, 1105–1120. 10.1037/0278-7393.31.5.1105 16248753

[B94] Peterson C. (1980). Recognition of noncontingency. J. Pers. Soc. Psychol. 38, 727–734. 10.1037/0022-3514.38.5.727

[B95] Phua D. H. Tan N. C. K. (2013). Cognitive aspect of diagnostic errors. Ann. Acad. Med. Singapore 42, 33–41. 23417589

[B96] Pineño O. Denniston J. C. Beckers T. Matute H. Miller R. R. (2005). Contrasting predictive and causal values of predictors and of causes. Learn. Behav. 33, 184–196. 10.3758/BF03196062 16075838

[B97] Pineño O. Miller R. R. (2007). Comparing associative, statistical, and inferential reasoning accounts of human contingency learning. Q. J. Exp. Psychol. 60, 310–329. 10.1080/17470210601000680 17366303 PMC1987335

[B98] Pronin E. Gilovich T. Ross L. (2004). Objectivity in the eye of the beholder: divergent perceptions of bias in self versus others. Psychol. Rev. 111, 781–799. 10.1037/0033-295X.111.3.781 15250784

[B99] Pronin E. Lin D. Y. Ross L. (2002). The bias blind spot: perceptions of bias in self versus others. Personal. Soc. Psychol. Bull. 28, 369–381. 10.1177/0146167202286008

[B100] Pronin E. Wegner D. M. McCarthy K. Rodriguez S. (2006). Everyday magical powers: the role of apparent mental causation in the overestimation of personal influence. J. Pers. Soc. Psychol. 91, 218–231. 10.1037/0022-3514.91.2.218 16881760

[B101] Rescorla R. A. Wagner A. R. (1972). “A theory of Pavlovian conditioning: variations in the effectiveness of reinforcement and nonreinforcement,” in Classical Conditioning II: Current Research and Theory, eds Black A. H. Prokasy W. F. (New York: Appleton-Century Crofts), 64–99.

[B102] Schmaltz R. Lilienfeld S. O. (2014). Hauntings, homeopathy, and the Hopkinsville Goblins: using pseudoscience to teach scientific thinking. Front. Psychol. 5:336. 10.3389/fpsyg.2014.00336 24860520 PMC4028994

[B103] Schwarz N. Sanna L. Skurnik I. Yoon C. (2007). Metacognitive experiences and the intricacies of setting people straight: implications for debiasing and public information campaigns. Adv. Exp. Soc. Psychol. 39, 127–161. 10.1016/S0065-2601(06)39003-X

[B104] Shaklee H. Mims M. (1981). Development of rule use in judgments of covariation between events. Child Dev. 52, 317–325.

[B105] Shang A. Huwiler-Müntener K. Nartey L. Jüni P. Dörig S. Sterne J. A. (2005). Are the clinical effects of homoeopathy placebo effects? Comparative study of placebo-controlled trials of homoeopathy and allopathy. Lancet 366, 726–732. 10.1016/S0140-6736(05)67177-2 16125589

[B106] Shanks D. R. (2007). Associationism and cognition: human contingency learning at 25. Q. J. Exp. Psychol. 60, 291–309. 10.1080/17470210601000581 17366302

[B107] Shanks D. R. Dickinson A. (1987). “Associative accounts of causality judgment,” in The Psychology of Learning and Motivation, Vol. 21, ed. Bower G. H. (San Diego, CA: Academic Press), 229–261.

[B108] Shanks D. R. Holyoak K. J. Medin D. L. (eds) (1996). The Psychology of Learning and Motivation: Vol. 34. Causal Learning. San Diego, CA: Academic Press.

[B109] Shanks D. R. Pearson S. M. Dickinson A. (1989). Temporal contiguity and the judgment of causality by human subjects. Q. J. Exp. Psychol. 41B, 139–159.

[B110] Shou Y. Smithson M. (2015). Effects of question formats on causal judgments and model evaluation. Front. Psychol. 6:467. 10.3389/fpsyg.2015.00467 25954225 PMC4404718

[B111] Shtulman A. Valcarcel J. (2012). Scientific knowledge suppresses but does not supplant earlier intuitions. Cognition 124, 209–215. 10.1016/j.cognition.2012.04.005 22595144

[B112] Singh S. Ernst E. (2008). Trick or Treatment: The Undeniable Facts about Alternative Medicine. New York: WW Norton & Company.

[B113] Stanovich K. E. (2011). Rationality and the Reflective Mind. New York: Oxford University Press.

[B114] Stephens A. N. Ohtsuka K. (2014). Cognitive biases in aggressive drivers: does illusion of control drive us off the road? Personal. Individ. Differ. 68, 124–129. 10.1016/j.paid.2014.04.016

[B115] Taylor S. E. Brown J. D. (1988). Illusion and well-being: a social psychological perspective on mental health. Psychol. Bull. 103, 192–210. 10.1037/0033-2909.103.2.193 3283814

[B116] Taylor S. E. Brown J. D. (1994). Positive illusion and well-being revisited: separating fact from fiction. Psychol. Bull. 116, 21–27. 10.1037/0033-2909.116.1.21 8078971

[B117] Vadillo M. A. Matute H. Blanco F. (2013). Fighting the illusion of control: how to make use of cue competition and alternative explanations. Univ. Psychol. 12, 261–270.

[B118] Vadillo M. A. Miller R. R. Matute H. (2005). Causal and predictive-value judgments, but not predictions, are based on cue-outcome contingency. Learn. Behav. 33, 172–183. 10.3758/BF03196061 16075837

[B119] Vadillo M. A. Musca S. C. Blanco F. Matute H. (2011). Contrasting cue-density effects in causal and prediction judgments. Psychon. Bull. Rev. 18, 110–115. 10.3758/s13423-010-0032-2 21327350

[B120] Waldmann M. R. Holyoak K. J. (1992). Predictive and diagnostic learning within causal models: asymmetries in cue competition. J. Exp. Psychol. Gen. 121, 222–236. 10.1037/0096-3445.121.2.222 1534834

[B121] Ward W. C. Jenkins H. M. (1965). The display of information and the judgment of contingency. Can. J. Psychol. 19, 231–241. 10.1037/h0082908 5824962

[B122] Wasserman E. A. (1990). “Detecting response-outcome relations: toward an understanding of the causal texture of the environment,” in The Psychology of Learning and Motivation, Vol. 26, ed. Bower G. H. (San Diego, CA: Academic Press), 27–82.

[B123] Wasserman E. A. Elek S. M. Chatlosh D. L. Baker A. G. (1993). Rating causal relations: role of probability in judgments of response-outcome contingency. J. Exp. Psychol. Learn. Mem. Cogn. 19, 174–188. 10.1037/0278-7393.19.1.174

[B124] Wasserman E. A. Kao S.-F. Van Hamme L. J. Katagari M. Young M. E. (1996). “Causation and association,” in The Psychology of Learning and Motivation, Vol. 34: Causal Learning, eds Shanks D. R. Holyoak K. J. Medin D. L. (San Diego, CA: Academic Press), 207–264.

[B125] Wasserman E. A. Neunaber D. J. (1986). College students’ responding to and rating of contingency relations: the role of temporal contiguity. J. Exp. Anal. Behav. 46, 15–35. 10.1901/jeab.1986.46-15 16812454 PMC1348253

[B126] Wheeler D. S. Miller R. R. (2008). Determinants of cue interactions. Behav. Process. 78, 191–203. 10.1016/j.beproc.2008.02.002 18355987 PMC2528875

[B127] White House Commission on Complementary and Alternative Medicine Policy. (2002). White House Commission on Complementary and Alternative Medicine Policy. Final report (WHCCAM Report). Available at: http://www.whccamp.hhs.gov/pdfs/fr2002_document.pdf [accessed March 28, 2015].

[B128] Willingham D. T. (2007). Critical thinking: why is it so hard to teach? Am. Educ. 31, 8–19. 10.3200/AEPR.109.4.21-32

[B129] Wiseman R. Watt C. (2006). Belief in psychic ability and the misattribution hypothesis: a qualitative review. Br. J. Psychol. 97, 323–338. 10.1348/000712605X72523 16848946

[B130] Yarritu I. Matute H. (2015). Previous knowledge can induce an illusion of causality through actively biasing behavior. Front. Psychol. 6:389. 10.3389/fpsyg.2015.00389 25904883 PMC4389369

[B131] Yarritu I. Matute H. Luque D. (2015). The dark side of cognitive illusions: when an illusory belief interferes with the acquisition of evidence-based knowledge. Br. J. Psychol. 10.1111/bjop.12119 [Epub ahead of print]. 25641547 PMC5024046

[B132] Yarritu I. Matute H. Vadillo M. A. (2014). Illusion of control: the role of personal involvement. Exp. Psychol. 61, 38–47. 10.1027/1618-3169/a000225 23948387 PMC4013923

[B133] Zhang Y. Risen J. L. Hosey C. (2014). Reversing one’s fortune by pushing away bad luck. J. Exp. Psychol. Gen. 143, 1171–1184. 10.1037/a0034023 23937176

[B134] Ziegler F. V. Tunney R. J. (2012). Decisions for others become less impulsive the further away they are on the family tree, PLoS ONE 7:e49479. 10.1371/journal.pone.0049479 23209580 PMC3509068

